# Development of occlusal canting identifying tool: verification, reliability and validation assessment

**DOI:** 10.1186/s12903-023-03802-5

**Published:** 2024-01-04

**Authors:** Hessah A. Alhuwaish, Khalid A. Almoammar, Abdulaziz S. Fakhouri

**Affiliations:** 1 Dental University Hospital, King Saud University Medical City, Riyadh, Saudi Arabia; 2 Department of Pediatric Dentistry and Orthodontics, College of Dentistry, King Saud University, PO Box: 60169, Riyadh, 11454 Saudi Arabia; 3 Department of Biomedical Technology, College of Applied Medical Sciences, King Saud University, Riyadh, Saudi Arabia

**Keywords:** Occlusal cant, Occlusal canting identifying tool, verification, laboratory validation

## Abstract

**Introduction:**

Occlusal cant (OC) is a malocclusion trait that lacks accurate clinical assessment methods. The occlusal canting identifying tool (OCIT) was invented and patented as a clinical tool to accurately identify and quantify the degree of maxillary OC. This study aimed to 1) develop a prototype of the OCIT, 2) verify the functionality of the OCIT and 3) assess the validity and reliability of the OCIT.

**Materials and methods:**

A patented OCIT design was revised, and the dimensions were finalized, followed by a three-dimensional conceptual prototype design that was reviewed and approved by the inventors. Verification was performed using a digital angle gauge to determine the accuracy of the bubble level as well as the angle between the bite plate and the protractor. For laboratory validation, 40 orthodontists measured the simulated OC at (0°, 2°, 4°, 6° and 8°) on five phantom heads using the OCIT. A reliability assessment of the tool was performed in three occasions by one orthodontist using the same laboratory settings.

**Results:**

The OCIT was prototyped from a medical-grade stainless steel alloy (316 L). Verification assessment revealed that the accuracy error of the bubble level (0.316° ± 0.028°) was statistically significant but clinically insignificant, while that of the angle between the bite plate and protractor (0.100° ± 0.050°) was statistically insignificant. Validation assessment showed high validity of the OCIT with no statistically significant difference between the OCIT and the reference values, having more errors in identifying smaller OC degrees compared to larger OC degrees. The intraclass correlation coefficient indicated the high reliability of the OCIT.

**Conclusion:**

The OCIT was verified and proven to be a valid and reliable clinical tool that accurately evaluates the degree of OC.

## Introduction

An occlusal cant (OC) is defined as the vertical alteration or upward/downward rotation of the occlusal plane (OP) in the transverse plane of one side over the other [1]. An OC may be related to dental or skeletal discrepancies, and on numerous occasions, they contribute to an underlying facial asymmetry [2]. Clinical assessments and radiographic evaluations are used to assess OC in the transverse plane [1, 3–5]. In the frontal view, one of the simplest clinical examination methods involves using a wooden tongue depressor to visualize the presence of a vertical discrepancy in the OP in relation to the interpupillary line [6]. A cant in the OP relative to the true horizontal plane can be determined by a posteroanterior (PA) cephalograms [3, 5, 7, 8]. In contemporary clinical assessment, the introduction of cone beam computed tomography (CBCT) and three-dimensional (3D) imaging techniques have aided in the detailed visualization of the craniofacial complex and occlusion and has gained popularity in clinical orthodontics [7, 8]. These assessment methods enhance orthodontics’ diagnosis and treatment planning processes by emphasizing specific dental and skeletal discrepancies, including facial asymmetries and OC [9–11].

Those diagnostic methods have several drawbacks. First, the clinical measurements rely on the subjective evaluation of the anatomical reference plane, which could affect the accuracy of the OC assessment in cases of facial asymmetry. Second, diagnostic measurements are performed by subjecting the patient to harmful ionizing radiation emitted during radiography. Therefore to overcome such drawbacks, the occlusal canting identifying tool (OCIT) was patented (patent no. US9987111, United States Patent Office) and invented as a clinical tool to accurately identify and quantify the degree of maxillary OC [12]. This adjustable tool can be used for all patients, including children and adults.

Although verification and validation are interrelated, they are independent terminologies, and each has distinct meaning in medical device development. Verification is defined as “the evaluation of whether or not a product, service, or system complies with a regulation, requirement or design specification” [13]. While validation is defined as “the degree to which the tool or method measures what it is meant to measure” [14].

The OCIT is highly necessary in clinical practice as a clinical tool that accurately evaluates the OC without subjecting the patient to ionizing radiation. This tool can be used in more than one discipline related to occlusion problems, including orthodontics, prosthodontics and maxillofacial surgery.

The aims of this study were to: 1) develop a prototype of the OCIT, 2) verify the functionality of the OCIT and 3) assess the validity and reliability of the OCIT.

## Materials and methods

Ethical approval (No. E-21-5905) for the study protocol was obtained from the Institutional Review Board (IRB) of King Saud University. The study was approved by and registered at the College of Dentistry, King Saud University (No. 0123).

### OCIT prototyping

The OCIT prototype (patent no. US9987111) was engineered, conceptualised, designed and manufactured in three phases (Dar Tec Engineering Consultants, Jeddah, Saudi Arabia) with the supervision of the inventors and the collaboration of the Technology Advancement and Prototyping Center (King Saud University). The following phases of OCIT development were implemented:

#### Phase 1 – conceptual design development

The first phase included a patent review to confirm the design, specific dimensions of the parts of the tool envisioned by the inventors and intended fabrication processes. It also involved confirming key features related to functionality, ergonomics and usability. Furthermore, we explored the features of the tool in terms of protractor rotation, adjustability, detachability and assembly. Then, 3D computer-aided design (CAD) files were generated for all the parts and assemblies of the tool (Solidworks, Chicago, United States of America).

#### Phase 2 – detailed design

The ultimate goal of this phase was to transform the conceptual design of the OCIT into a detailed design before manufacturing. In this phase, the inventors periodically reviewed the design, and a final conceptual layout was developed. The final materials were evaluated and selected. Finally, 3D CAD models were updated accordingly for all components and assemblies (Solidworks, Chicago, United States of America).

#### Phase 3 – functional prototyping

In this phase, a functional prototype was manufactured from the intended materials, assembled, inspected and mechanically assessed. The functional prototype was manufactured from a medical-grade stainless-steel alloy (316 L). Subtractive manufacturing was performed using a metal laser cutting machine (Stylencnc, Shandong, China) and a conventional lathing machine (Knuth, Guangzhou, China).

### Verification assessment of the OCIT

**Fig. 1:**
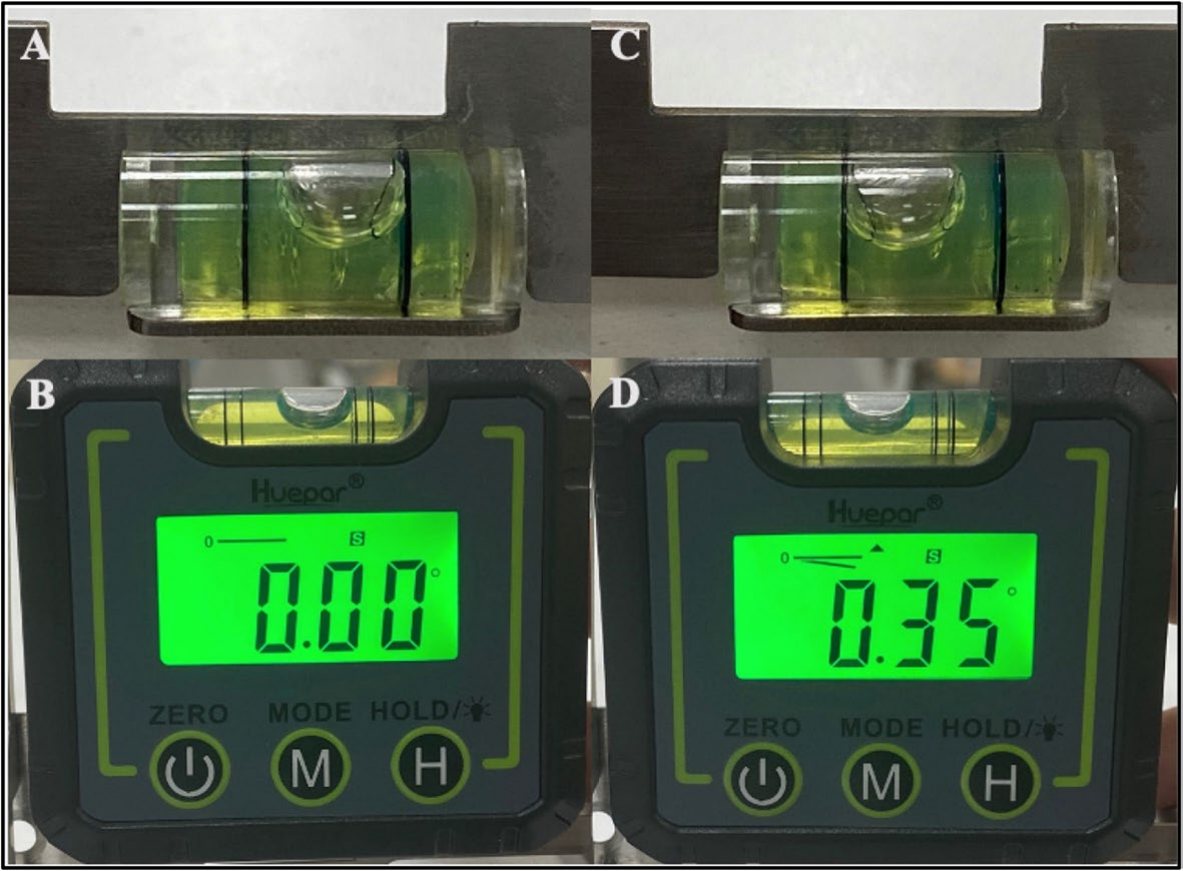
Verification assessment of the OCIT by detecting the accuracy of the bubble level, **A** the bubble in touch with the right mark, **B** reading of the digital angle gauge, **C** the bubble in touch with the left mark, **D** reading of the digital angle gauge

The prototype was verified using two methods: first, by determining the accuracy of the bubble level attached to the OCIT and second, by evaluating the angle between the bite plate and the protractor. To determine the accuracy of the bubble level, a calibrated digital angle gauge (Huepar^®^, Levelsure Technology Co., Ltd., Zhuhai, China) was used. The digital angle gauge was placed on a flat surface, tared to 0° alongside the OCIT. The anterior horizontal body of the OCIT was adjusted until the bubble touched the right mark of the bubble level (Fig. 1A). At this point, the digital angle gauge was positioned at the upper border of the anterior horizontal body and tared to 0° again (Fig. 1B). Then, the anterior horizontal body was rotated clockwise until the bubble touched the left mark of the bubble level (Fig. 1C). The digital angle gauge was placed again at the anterior horizontal body of the OCIT, and the angle was calculated (Fig. 1D).

**Fig. 2:**
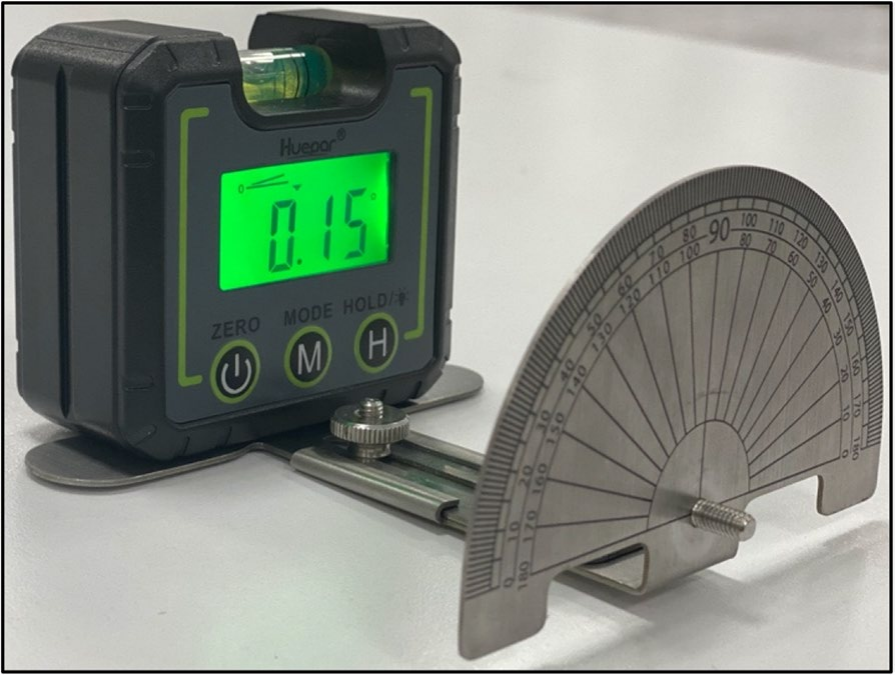
Verification assessment of the OCIT by evaluating the relationship between the bite plate and the protractor

The second verification assessment was performed by evaluating the angle between the bite plate and the protractor to determine if there were any angular discrepancies between them. This rotatable part was disassembled from the anterior horizontal body and placed on a flat surface, and the digital angle gauge was placed on the bite plate to assess the correlation between 0° on the protractor and the bite plate (Fig. 2). The same researcher repeated both verification assessments on three separate occasions to verify the components of the OCIT.

### Assessment of the validity and reliability of the OCIT

#### Model preparation and verification

**Fig. 3:**
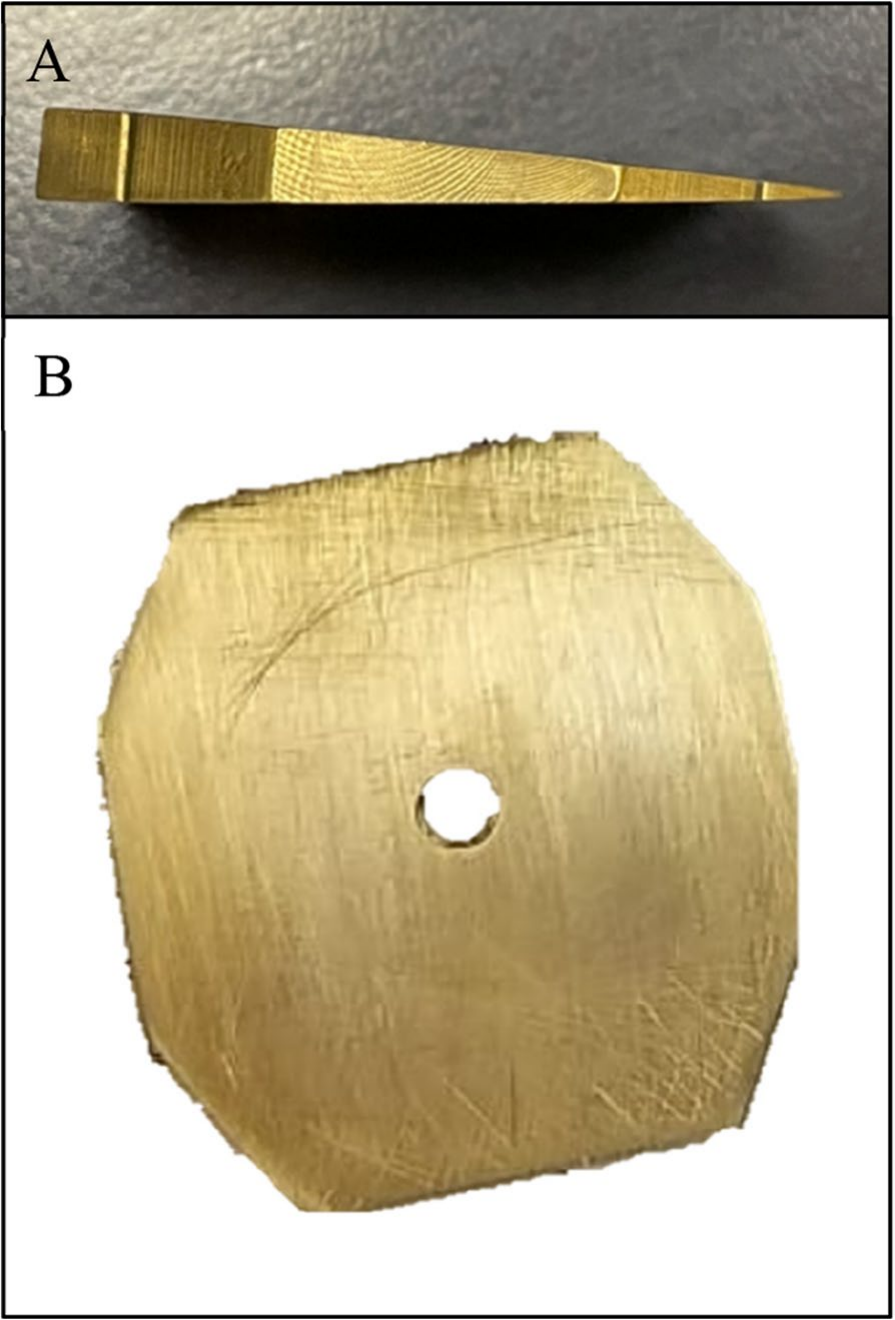
Fabricated metal wedge, **A **side view, **B** Top view

**Fig. 4:**
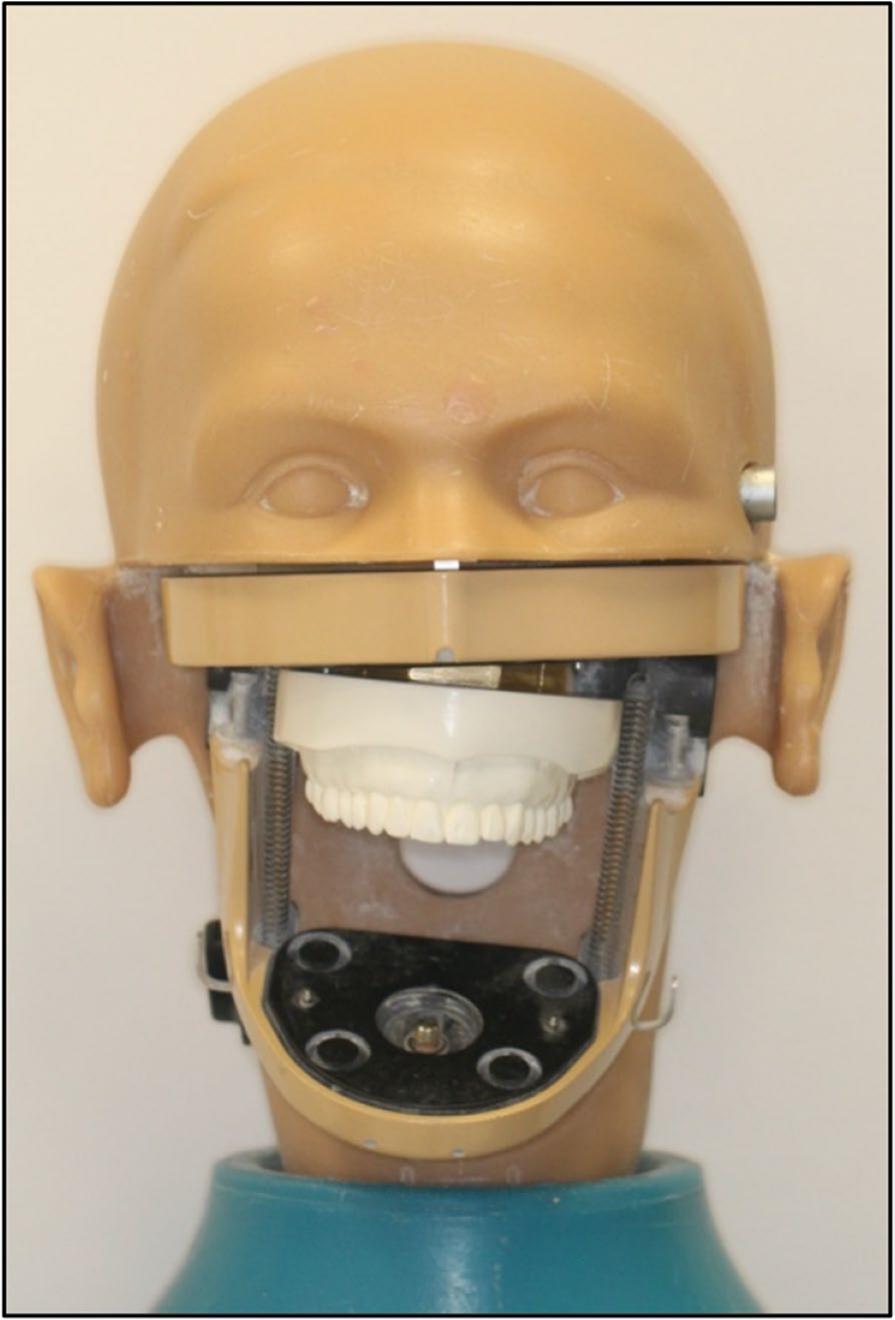
Final prepared phantom head

**Fig. 5:**
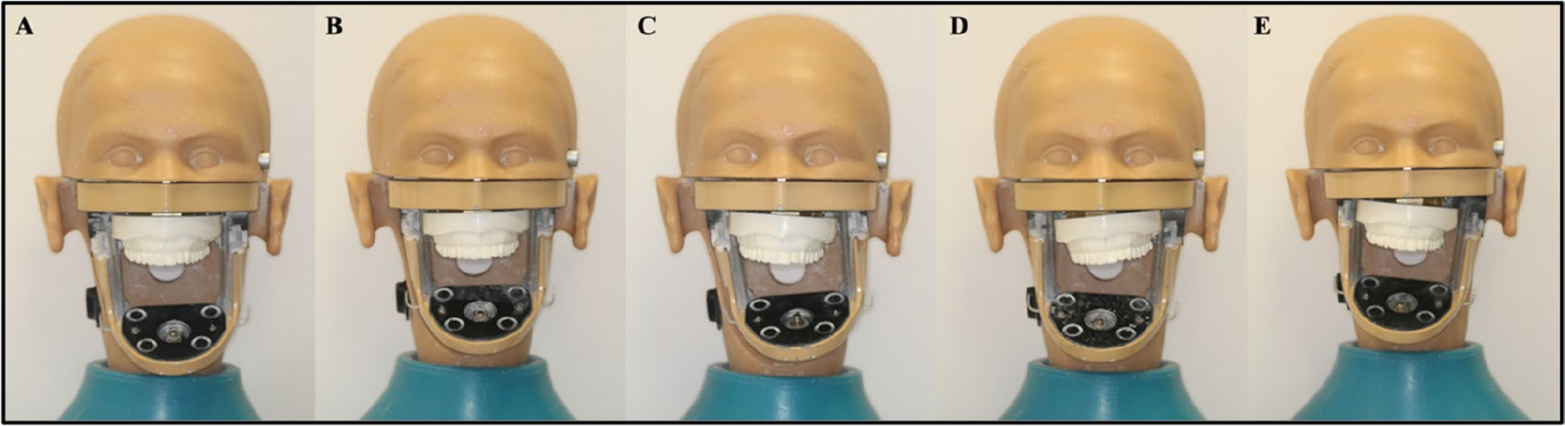
Preparation of the phantom heads, **A** Control - 0° OC, **B** 2° OC to the right, **C** 4° OC to the left, **D** 6° OC to the right, **E** 8° OC to the left

**Fig. 6:**
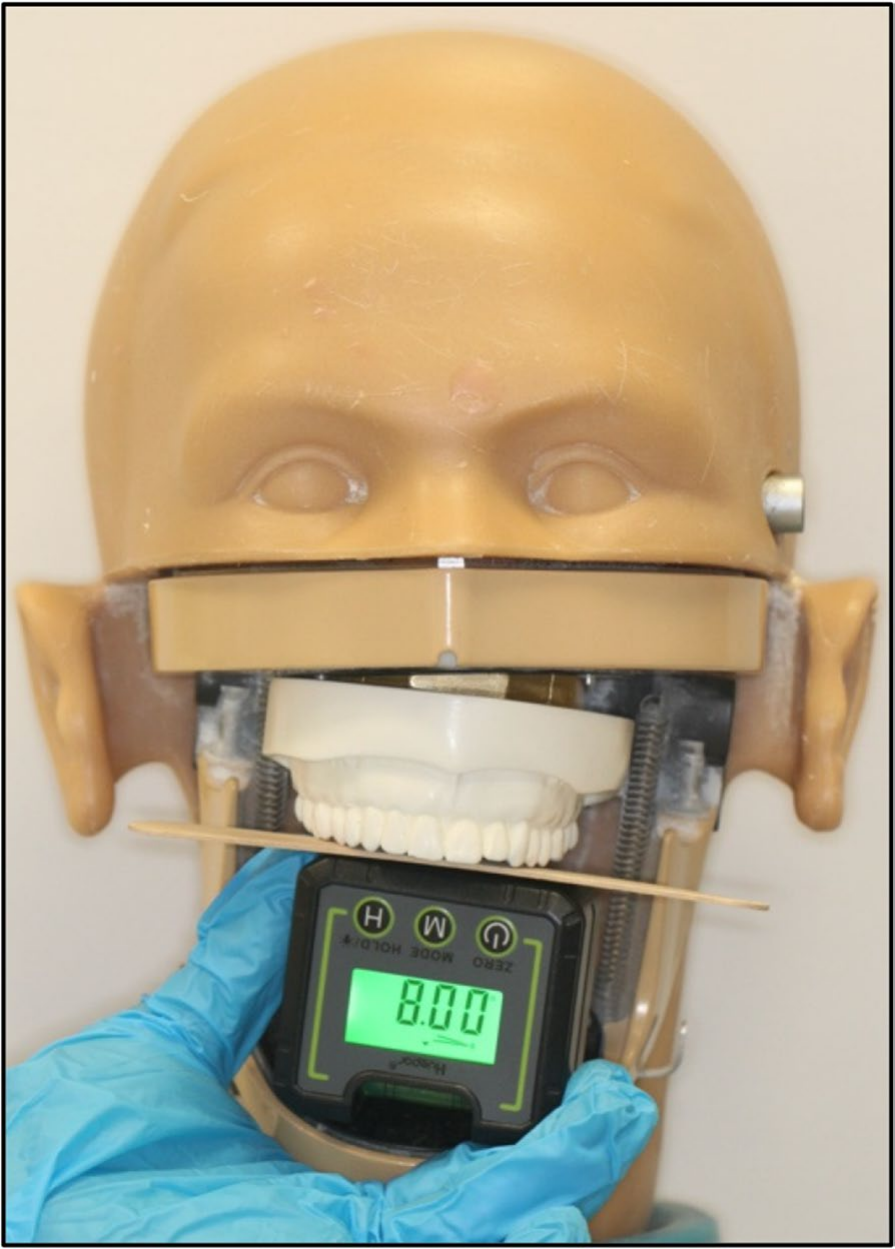
Illustration of the verification assessment method of the phantom heads

The OC was simulated on phantom heads at the Prosthetics Laboratory, College of Dentistry, King Saud University. Five phantom heads (KaVoKerr, Berlin, Germany) were used: one serving as a control and the remaining four as models for occlusal cant. The heads of the four phantom models were prepared using the following steps. First, the simulated OC was represented by four customised metallic wedges with predetermined angles (2°, 4°, 6°, and 8°) that were randomly allocated equally as either left- or right-sided canted wedges. The wedges were fabricated with a screw thread that matched the size of the stabilizing screw on the phantom head (Fig. 3). Next, an upper jaw model (Nissin Dental Products, Kyoto, Japan) was duplicated using an alginate impression (Integra™ Alginate, Berlin, Germany) and then poured with a plaster material (Plaster dental material Snow White™, KaVoKerr, Berlin, Germany). The base of the plaster model was glued flush to the tilted side of the wedge using Zapit Super Glue (Super Glue Corporation, Ontario, CA, United States), and all excess was removed. The whole unit was then attached to the phantom head by passing the main stabilizing screw through the created screw thread (Fig. 4). The control phantom head was prepared following the same steps, except for the fabrication of a rectangular metal base instead of a wedge to produce a 0° angle for the OC (Fig. 5). To verify the accuracy of the fabricated wedges and duplicated study models, a wooden tongue plate was placed at the occlusal surface of the upper premolars at each phantom head to create a flat surface, and a digital angle gauge was used. The verification process was performed by the same researcher on three separate occasions at 2 weeks intervals (Fig. 6).

#### Validation process

Forty orthodontists from the Dental University Hospital of King Saud University with at least 3 years of experience were recruited, and written consent forms were obtained. Each evaluator measured the five prepared models. The use of the device was demonstrated to each expert, where the phantom head was first kept upright, the tool was adjusted based on the facial width and depth of the phantom head, and the bubble level was used to orient the anterior horizontal body exactly parallel to the horizontal plane using the bubble level attached to the OCIT. For standardisation, the tool was initially adjusted by the researcher, and the expert supported the bite plate in the upper OP at the premolar region. The protractor would accordingly rotate in the direction of the OP tilt, and the expert would detect the degree of canting if present. The measurements provided by each evaluator were recorded on an Excel spreadsheet (Microsoft Office Excel 2019, Microsoft Corporation, Redmond, WA, USA). For inter- and intra-examiner reliability, 10 orthodontists were randomly selected to repeat the experiment after 2 weeks.

#### Reliability assessment

An orthodontist with 3 years of experience was randomly selected from the Dental University Hospital of King Saud University to assess the reliability of the tool. Following the same steps as that performed in the validation process, the assigned orthodontist was requested to measure the OC of the five phantom heads using the OCIT on three separate occasions at 2 weeks intervals. This orthodontist was not involved in the validation process.

### Statistical analysis

All data were analyzed using the Statistical Package for the Social Sciences software (SPSS) version 26.0 (IBM Inc., Chicago, IL, USA). Descriptive statistics (means, standard deviations, and frequencies) were used to describe all quantitative variables. Statistical significance was set at *P* ≤ 0.05. A one-sample *t*-test was used to verify and validate the OCIT, while the intraclass correlation coefficient (ICC) was used to test the inter- and intra-examiner reliabilities of the participants in the validation process as well as the reliability of the OCIT.

## Results

### OCIT prototyping

**Fig. 7:**
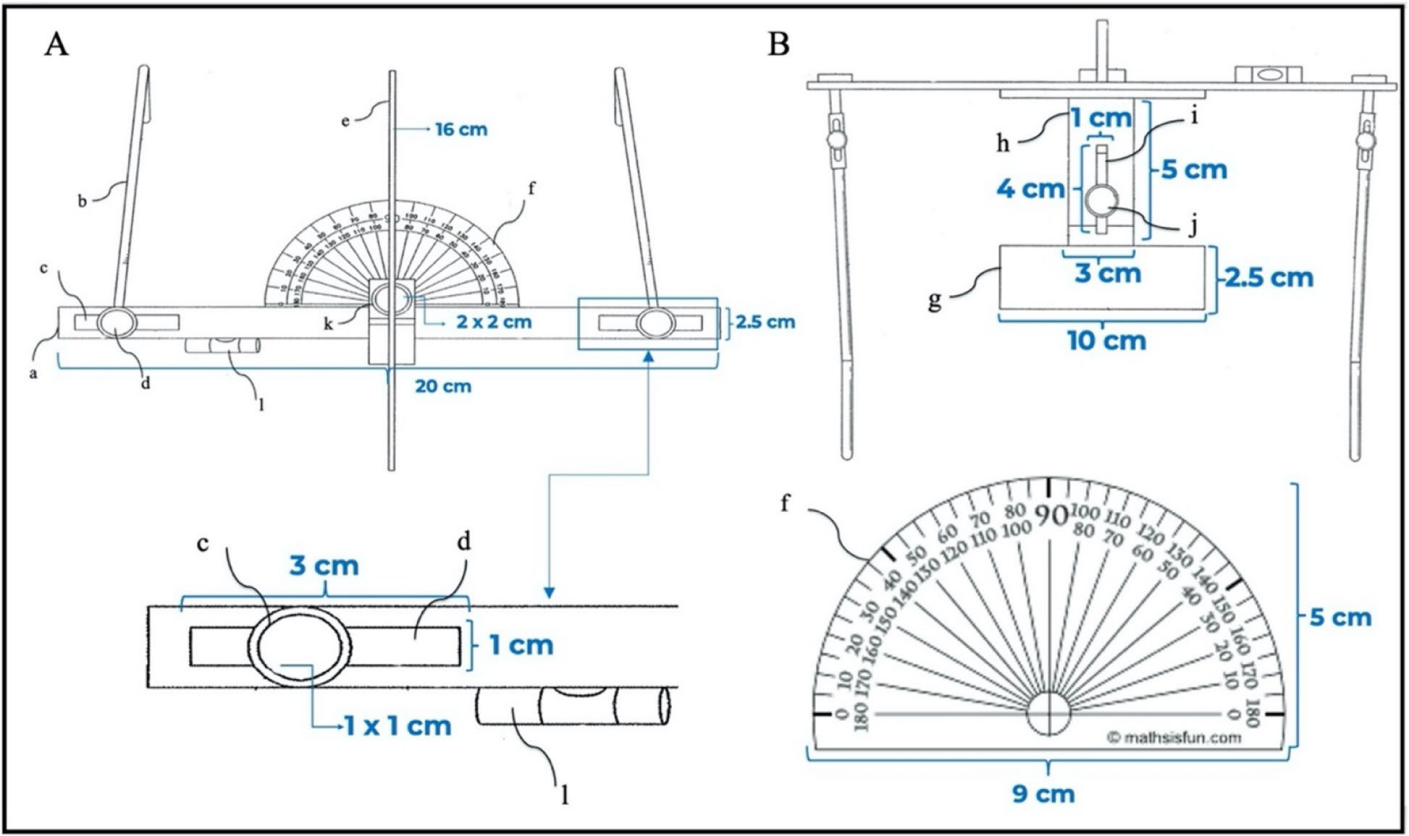
Design and measurements of the OCIT patent, **A** Front view, **B** Top view; *a. anterior* horizontal body, b. side arm, c. side arm sliding slot, d. side arm thumbscrew, e. vertical arm, f. protractor, g. bite plate, h. sliding sheet, i. sliding sheet slot, j. bite plate thumbscrew, k. main thumbscrew, l. bubble levelling device

The OCIT conceptual design included an initial design composed of several parts that could be assembled into a single device (Fig. 7A, B). The OCIT has a U-shaped frame consisting of an anterior horizontal body (20 cm width × 2.5 cm height) and two parallel adjustable side arms (15 cm in length) attached at 90° to the anterior horizontal body. These arms can be adjusted medio-laterally to fit the face of most patients via two-sided thumbscrews (1 cm height × 1 cm width). The side arms passed through two horizontal slots (3 cm width × 1 cm height) at each side of the anterior horizontal body. Anterior to the anterior horizontal body is a vertical arm (16 cm height × 2 cm depth) corresponding to the facial midline and a bubble level representing the true horizontal plane, which serves as the non-anatomical reference plane to be used for OC assessments. The movable components of the tool are attached behind the anterior horizontal body. They act as one unit, consisting of a protractor and an adjustable sliding sheet with a bite plate. The protractor (9 cm width × 5 cm height) is the measuring component, which is attached to the bite plate (10 cm width × 2.5 cm height), which rests at the occlusal surface of the upper arch. The distance between the bite plate and protractor was adjustable using a sliding sheet (3 cm width × 5 cm height) through a slot (1 cm width × 4 cm height) and stabilized with the thumbscrew (1 cm width × 1 cm height). The movable components of the protractor, sliding sheets and biting plate rotated axially as one unit around the main thumbscrew (2 cm height × 2 cm width) to measure OC in degrees.

**Fig. 8:**
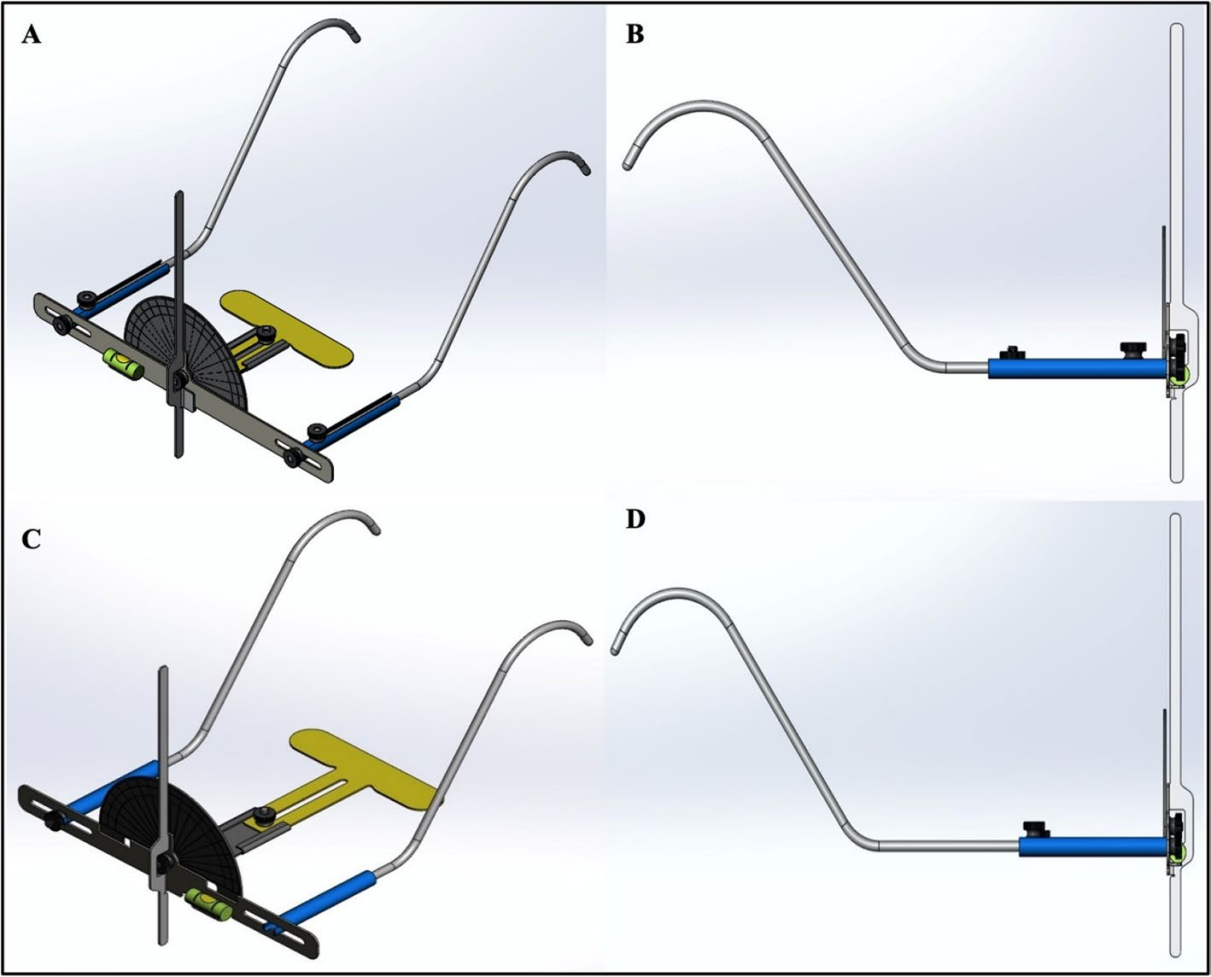
3D CAD model of the occlusal cant identifying tool, **A** isometric view of the initial 3D CAD model, **B** side view of the initial 3D CAD model, **C** isometric view of the final 3D CAD model, **D** side view of the final 3D CAD model

A 3D CAD model rendering was performed to reflect the detailed design and prepare for the manufacturing process. The initial 3D CAD files of the tool parts and assemblies were presented by DarTec company to the inventors (Fig. 8A, B) and mediated by the Technology Advancement and Prototyping Center. All adjustments and modifications were updated in the final 3D CAD file (Fig. 8C, D). The main difference between the initial and final 3D CAD files is the design of the side arms, which was revised from screw-dependent to flexible arms.

**Fig. 9:**
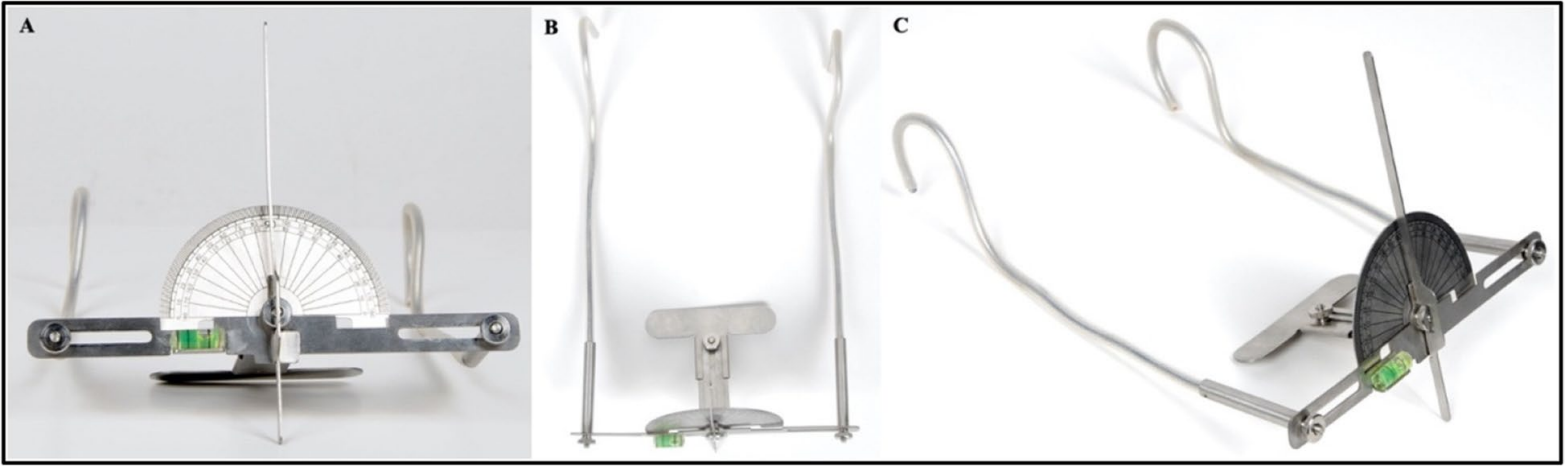
Final functional prototype of OCIT, **A** front view, **B** top view, **C** side view

The functional prototype of the OCIT was developed from a medical-grade stainless steel alloy (316 L) (Fig. 9). The OCIT can be used by adjusting the distance between the bite plate and anterior horizontal body, the flexible side arms to fit the distance between the patient’s two ears and the end of each flexible side arm to fit firmly around the patient’s two ears. The orientation of the anterior horizontal body should be perfectly parallel to the horizontal plane using a bubble-levelling device. Once OC is detected by the biting plate, the protractor axially rotates in the direction of canting, and the upper border of the anterior horizontal body is used to determine the degree of canting.

### Verification of OCIT

To verify that the bubble level on the OCIT accurately indicates the true horizontal plane (0°), verification assessment using the angle digital gauge showed that the accuracy of the bubble level had an error margin of 0.316° ± 0.028° (*n* = 3) (mean ± SD), which was significantly different from 0° (*P* ≤ 0.05). To further verify the utility of the OCIT and confirm the accuracy of the protractor in showing the correct angle of the bite plate, the angle between the bite plate and the protractor was measured, which was 0.100° ± 0.050° (mean ± SD) in the counterclockwise direction and was statistically insignificantly different from 0° (*P* ≤ 0.05). Table 1 summarises the results of the verification tests.

**Table 1:** Verification assessment results of the OCIT prototype

Verification parameters	n	Mean ± SD (°)	Std. Error	*t*-test	df	^a^*P*-value	95% Confidence Interval of the Difference	
							Lower Bound	Upper Bound
Accuracy error of bubble level	3	0.316 ± 0.028	0.016	19.00	2	0.003^**^	0.245	0.388
Accuracy error of angle between bite plate and protractor	3	0.100 ± 0.050	0.028	3.46	2	0.074	−0.024	0.224

^a^*P*-Value * = *P* ≤ 0.05, ** = *P* ≤ 0.01, *** = *P* ≤ 0.001

### Verification of phantom head models

The verification assessment of each phantom head using the calibrated angle digital gauge showed that the accuracy of the fabricated metal wedges was acceptable, as all measurements were statistically insignificant (*p* ≤ 0.05) compared to the reference value of each phantom head (Table 2).

**Table 2:** Verification assessment results of the phantom head models

Phantom Head	n	Ref. value (°)	Mean ± SD (°)	Std. Error	*t*-test	df	^a^*P*-value	95% Confidence Interval of the Difference	
								Lower Bound	Upper Bound
1	3	0.00	0.003 ± 0.005	0.003	1.00	2	0.423	−0.065	0.158
2	3	2.00	2.046 ± 0.045	0.026	1.793	2	0.215	−0.065	0.158
3	3	4.00	4.056 ± 0.051	0.029	1.913	2	0.196	−0.070	0.184
4	3	6.00	6.043 ± 0.040	0.023	1.857	2	0.204	−0.057	0.143
5	3	8.00	8.060 ± 0.052	0.030	1.964	2	0.188	−0.071	0.191

^a^*P*-Value * = *P* ≤ 0.05, ** = *P* ≤ 0.01, *** = *P* ≤ 0.001

### Validation and reliability assessment of OCIT

#### Validation process

Due to the novelty of the newly patented tool used in our study, it was not possible to base the sample size on a previous study. A pilot study was conducted consisting of 12 participants and the sample size estimation based on a power of 0.9 and a *P*-value of 0.05 for a medium effect size of 0.5 confirmed that the required number of participants to be enrolled was 40 orthodontists [15]. The inter- and intraexaminer reliabilities among the participants showed a high ICC value of 0.95 and 0.98, respectively. The overall responses of the participants showed a high level of validity of the newly developed OCIT, and all the records on the five phantom heads showed no statistically significant difference compared with the reference values on the metal wedges on each phantom head (Table 3). The means of the measurements of the OCIT on the phantom heads showed overall smaller values than the reference values. The responses showed that the smaller the angle, the more difficult it was to identify OC using the OCIT and vice versa.

**Table 3:** Validation assessment results of the OCIT

Phantom Head	n	Ref. value (°)	Mean ± SD (°)	Std. Error	*t*-test	df	^*a*^*P*-value	95% Confidence Interval of the Difference	
								Lower Bound	Upper Bound
1	40	0.00	0.075 ± 0.266	0.042	1.778	39	0.083	−0.010	0.160
2	40	2.00	1.825 ± 0.635	0.100	−1.740	39	0.090	−0.378	0.028
3	40	4.00	3.875 ± 0.515	0.081	−1.533	39	0.133	−0.289	0.039
4	40	6.00	5.950 ± 0.316	0.050	−1.000	39	0.323	−0.151	0.051
5	40	8.00	7.975 ± 0.276	0.043	−0.572	39	0.570	−0.113	0.063

^a^*P*-Value * = *P* ≤ 0.05, ** = *P* ≤ 0.01, *** = *P* ≤ 0.001

#### Reliability process

The ICC indicated a high level of reliability of the newly developed OCIT (0.85), as any value more than 0.8 is considered a high reliability value.

## Discussion

To date, no valid clinical tool has been designed to accurately measure the degree and direction of OC clinically. This study developed a prototype of the OCIT, verified its functionality, and assessed its validity and reliability. The proposed null hypothesis was that OCIT is not valid nor reliable when tested on simulated OC.

The patented OCIT (no. US9987111) was revised, and the dimensions were finalised, followed by a 3D conceptual prototype design that was reviewed and approved by the inventors. The inventors changed the adjustability mechanism of the side arms from screw-dependent to flexible arms to reduce the OCIT weight and complexity of the assemblies. Subsequently, the OCIT was manufactured from a medical-grade stainless steel alloy (316 L). It is the most commonly used alloy in medicine owing to its superior mechanical properties and biocompatibility [16, 17]. The nickel component provides good corrosion resistance properties, which is critical for the longevity of medical tools and instruments [18]. The aforementioned steps, procedures and material considerations are critical to warrant a tool with appropriate design and accurate dimensions.

The presence of OC is widely recognized as a significant flaw that can have a detrimental impact on facial attractiveness, leading to disruption of the overall harmony and balance of the face [19]. This tool is intended to provide an accurate clinical assessment of the OC in terms of its angle and direction. A clinician can perform an objective clinical evaluation of the cant prior to resorting to a plain radiograph or the more sophisticated CT. The OCIT can measure any degree of canting in any direction using a protractor and a bite plate. It can be used universally and is appropriate for all patients because of the adjustable sheets related to the occlusal plate and vertical arms.

Validity evaluation of any given tool or device assesses the accuracy of the achieved outcome using the tool with respect to an existing theory or known fact [20]. Ideally, a gold standard for validity should be used to compare the accuracy of the prepared tool with the standard method of clinical assessment. However, following the requirements of the Food and Drug Administration (FDA), a pre-clinical implementation for a newly developed tool is mandatory for FDA approval of medical devices [11]. Hence, to conduct pre-clinical or initial procedures to assess the verification, reliability and validity of the OCIT, a simulated phantom head was prepared to mimic a clinical scenario in a laboratory setting.

Verification attests to the accuracy of the critical components and the functionality of the OCIT. The margin of error found in the angle between the bite plate and protractor was not considered because the accuracy error was statistically insignificant, whereas the margin of error, representing the accuracy error of the bubble level was statistically significant. The estimated margin of error in angular measurements when CBCT images was compared with actual anatomical measurements was 0.38°, which is considered clinically acceptable [21]. Because the OCIT focuses on the angular measurement of the OC, the bubble level error is not clinically significant; therefore, it is negligible. Such an insignificant accuracy error confirms that the developed OCIT is highly accurate for OC identification.

The five phantom heads had models assembled with different angulations: 0°, 2°, 4°, 6°, and 8°. The rationale of the selected angles was to have a range of OC with equal intervals. It was also important to verify the accuracy of the fabricated wedges of the phantom head model, which may influence the accuracy of the measurements performed by the OCIT. The calibrated digital gauge showed that the metal wedges designed to mimic cants in the prepared models were accurate and statistically insignificantly different from the intended angle of the OC.

The main objective of this study was to assess the validity of the proposed tool; in other words, to assess whether the tool accurately measures what it is intended to measure [22]. In the design of this study, the prepared models and wedges resembling OC, which were assembled into a phantom head, were used as a platform to mimic a living patient. There were no statistically significant differences in the measurements performed on phantom heads using the OCIT compared to the actual cant created by the wedges. The responses showed that the smaller the angle, the more difficult it is to identify OC using OCIT and vice versa. These high validity outcomes are supported by the high repeatability of the measurements when inter- and intra-examiner reliabilities were assessed. ICC testing showed a highly reliable OCIT, which is another element for new tool assessment.

The OCIT has been shown to accurately measure OC at various angles. It will be appropriate for occlusal examinations in orthodontic, prosthodontic, and maxillofacial surgery. This will enable clinicians to reach a diagnosis in the orientation of the OP without the need for radiographic images, except in circumstances in which surgical intervention is required. 3D CBCT is required to accurately plan surgical movements in three planes of space [23].

Further, this device is lightweight, easily assembled, and used. The bite plate is the only component that requires sterilization via autoclaving. However, the tool has the disadvantage of size, as it is bulky, requiring the burden of space in storage. Moreover, the device must be stored in a protected enclosure to avoid bending, dents, or damage that might affect the accuracy of the measurements.

It is important to acknowledge that the current assessment stage is considered pre-clinical. To further validate and generalize the findings, it is highly recommended to conduct a clinical study involving live patients. This would provide a more comprehensive evaluation of the accuracy and usability of the OCIT in a real-world setting.

Looking ahead, it would be beneficial to explore the possibility of replacing the protractor component with a digital angle gauge. This advanced technology would allow for more precise measurements of small degrees of OC, leading to increased accuracy in assessing the condition.

The present study attempted to pre-clinically assess the newly invented OCIT, while the next recommended step was to clinically assess the tool in a clinical setting. Given the outcomes of the present study, the OCIT is useful and accurate for measuring OC before resorting to more sophisticated radiographic images.

## Conclusions

OCIT was verified and proven to be a valid and reliable clinical tool that provides an accurate clinical assessment of OC in terms of angle and direction.

## Data Availability

The data will be available upon request by contacting the corresponding author through e-mail communication.
